# Supplementary material for: A Comprehensive Analysis of Cutaneous Melanoma Patients in Greece Based on Multi-Omic Data
Source: Cancers (Basel). 2023 Jan 28;15(3):815. doi: 10.3390/cancers15030815 (PMC9913631; doi:10.3390/cancers15030815)
Supplement: Supplementary file 1 [file cancers-15-00815-s001.zip › Figures S1-S4.pdf]

Supplementary Figures

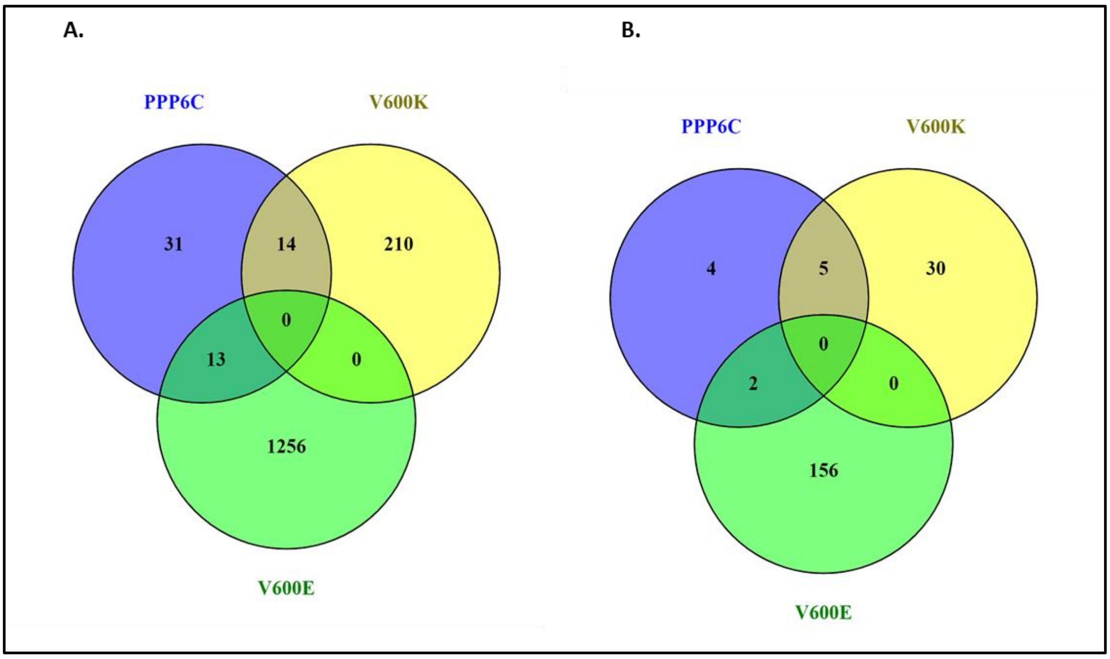

Supplementary Figure S1. *Venn diagram showing the proportion of BRAF V600E and V600K cases carrying also the PPP6C R264C mutation in melanoma samples from GENIE (A) and PanCancer TCGA (B) project.*

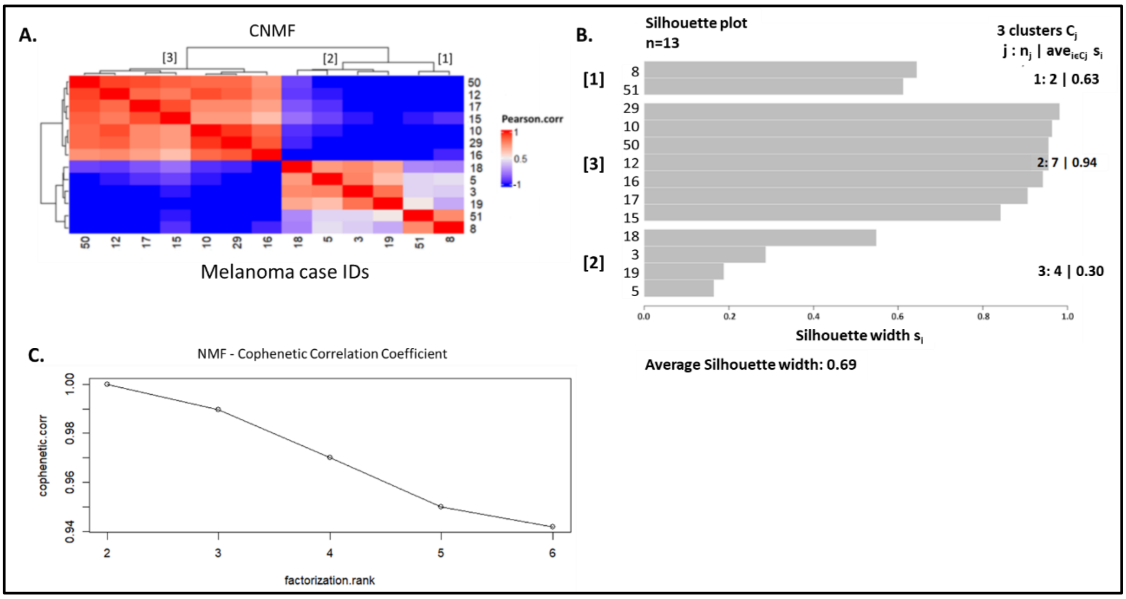

Supplementary Figure S2. *Consensus non-negative matrix factorization (CNMF) of top 1500 most highly variable genes of thirteen in-house melanoma patients.* ***A.*** *Feature selection was based on the 1500 most variant Median Absolute Deviation (MAD) log_2CPM gene expression values. Pearson correlation coefficient was used as a measure for the similarity matrix. Red*

and blue colours depict high and low similarity between samples, respectively. With [1], [2], and [3] are denoted the three consensus clusters, while the cluster membership is documented both at the bottom and on the right of this panel. ***B.*** *Silhouette plot of the three clusters derived from CNMF. A value near +1 indicates that the sample is well matched, while 0 indicates that the sample is on or very close to the decision boundary between two neighbouring clusters and negative values indicate that those samples might have been assigned to the wrong cluster. Each horizontal line represents a sample in the Silhouette plot. The length of the line denotes the silhouette width of the sample.* ***C.*** *Plot of Cophenetic correlation coefficients for r=2, 3, 4, 5, 6. This graph indicates the stability of the clusters obtained from CNMF for different values of the factorization rank. The choice of three clusters r = 3 was the optimal compared to lower ranks.*

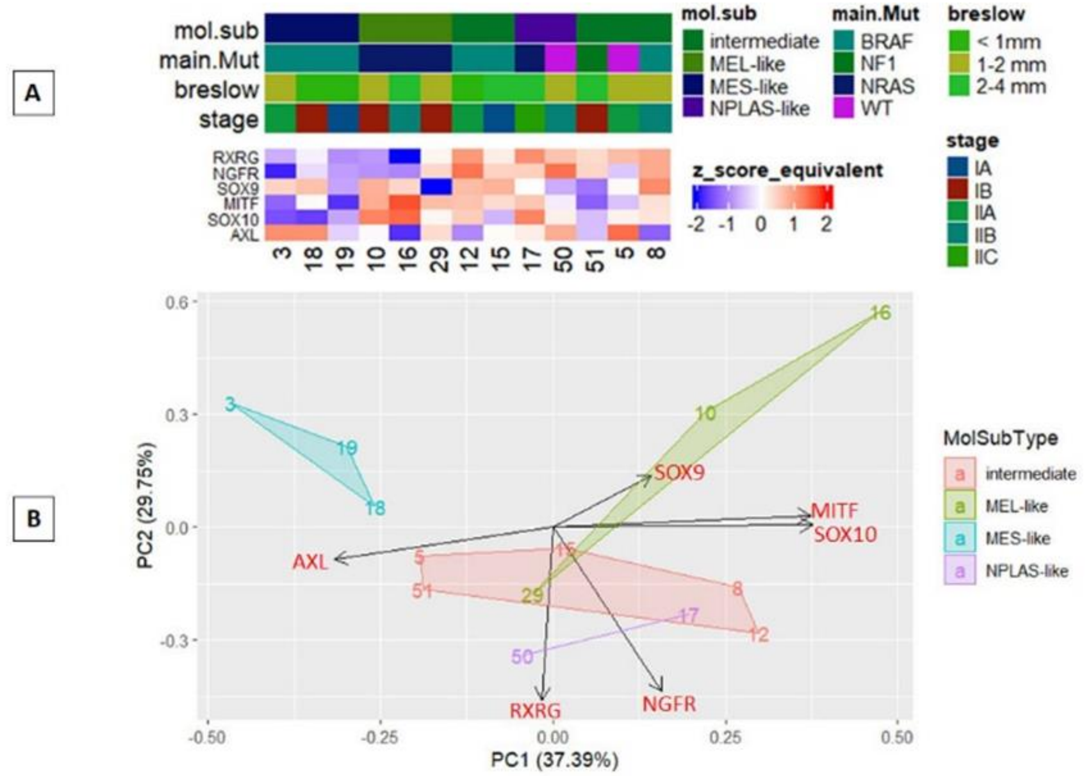

Supplementary Figure S3. *(A). Heatmap of the normalized gene expression values of some key cell-state regulatory genes (such as NGFR, RXRG, SOX9, SOX10, MITF and AXL) across the thirteen melanoma tumors. Rows in heatmap have been reordered by the method of hierarchical clustering (complete method, Euclidean distance), whereas columns (melanoma cases) are ordered based on the respective molecular subtype. Samples with high gene expression levels are marked in red and samples with relatively low gene expression levels are marked in blue. Clinical and extra molecular information (mutated driver genes) of samples is added on top as colour bars. (B). PCA plot of 13 melanoma cases for the principal components PC1 and PC2 based on the normalized gene expression values of key cell-state regulatory genes NGFR, RXRG, MITF, SOX10, SOX9 and AXL. Each sample is labeled with its unique identification number and samples of the same molecular subtype (MolSubType) are grouped into frames with different colours, where intermediate MolSubType denotes the non-MEL, non-MES samples. The PCA loadings (gene expression vectors) that discriminate our samples are indicated by arrows. The angle of each PCA loading in relation to x- or y-axis represents the*

contribution of each feature (gene) in the direction of the Principal Components (PCs) where it contributes. The length of this arrow depicts the strength of the contribution of the feature in that direction. NGFR: Nerve Growth Factor Receptor; RXRG: Retinoid X Receptor Gamma; SOX9: SRY-Box Transcription Factor 9; SOX10: SRY-Box Transcription Factor 10; MITF: Melanocyte Inducing Transcription Factor; AXL: AXL receptor tyrosine kinase.

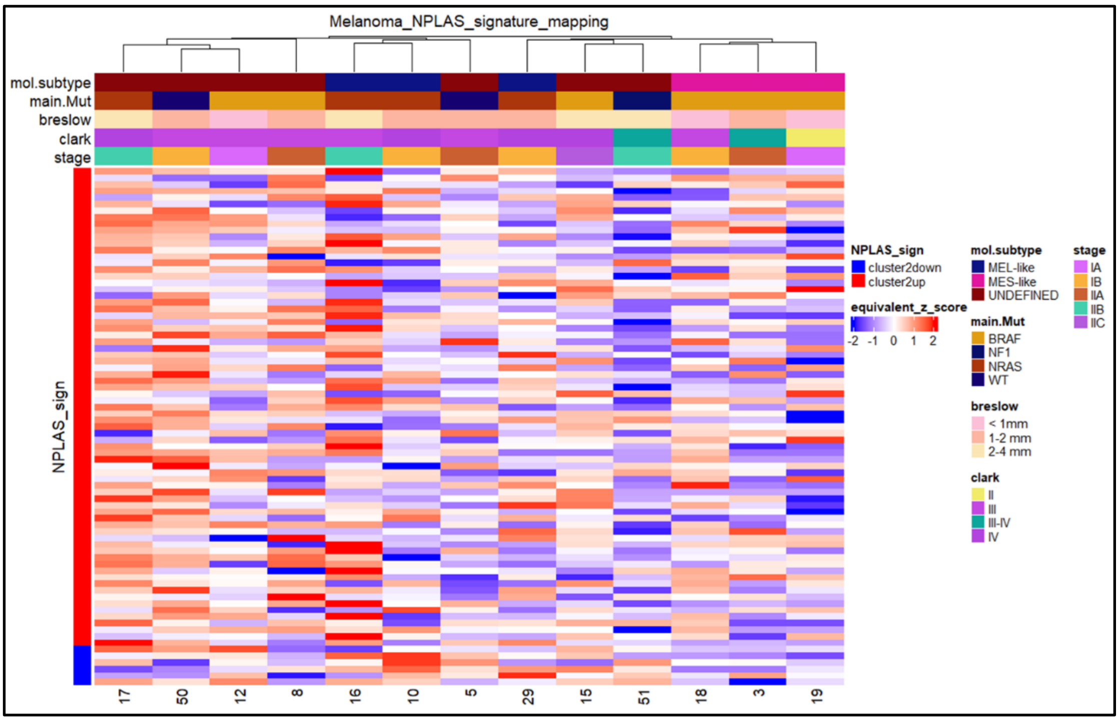

Supplementary Figure S4. *Heatmap of the relative mRNA expression of NPLAS-associated genes in melanoma samples (n=13) with sample cluster assignments by hierarchical clustering. Hierarchical clustering in columns (melanoma cases) based on Pearson correlation with average-linkage between samples. The NPLAS gene signature (NPLAS_sign) is composed of 78 over- (red vertical bar on the left) and 6 under-expressed (blue vertical bar on the left) genes as defined in Andrews et al. 2022. Gene expression values were z-transformed and coloured red for high expression and blue for low expression, as indicated in the scale bar. Clinical (breslow;clark and stage) and molecular information (mutated driver genes) of samples is added on top as colour bars.*
